# Safety and efficacy of peptide receptor radionuclide therapy with ^177^Lu-DOTA^0^-Tyr^3^-octreotate in combination with amino acid solution infusion in Japanese patients with somatostatin receptor-positive, progressive neuroendocrine tumors

**DOI:** 10.1007/s12149-021-01674-9

**Published:** 2021-09-17

**Authors:** Noritoshi Kobayashi, Shoko Takano, Kenichi Ito, Madoka Sugiura, Matsuyoshi Ogawa, Yuma Takeda, Naoki Okubo, Akihiro Suzuki, Motohiko Tokuhisa, Tomohiro Kaneta, Daisuke Utsunomiya, Masaharu Hata, Tomio Inoue, Makoto Hosono, Seigo Kinuya, Yasushi Ichikawa

**Affiliations:** 1 Department of Oncology, Oncology Division, Yokohama City University Graduate School of Medicine, 3-9, Fuku-ura, Kanazawa-ku, Yokohama, 236-0004 Japan; 2 Department of Radiation Oncology, Yokohama City University Graduate School of Medicine, Yokohama, Japan; 3 Department of Radiology, Yokohama City University Graduate School of Medicine, Yokohama, Japan; 4 Radiation Department, Yokohama City University Hospital, Yokohama, Japan; 5 Department of Diagnostic Image Analysis, Graduate School of Medicine, Tohoku University, Sendai, Japan; 6 Advanced Medical Center, Shonan Kamakura General Hospital, Kamakura, Japan; 7 Department of Radiology, Faculty of Medicine, Kindai University, Higashi-osaka, Japan; 8 The Japanese Society of Nuclear Medicine, Tokyo, Japan

**Keywords:** Peptide receptor radionuclide therapy, Neuroendocrine tumors, ^177^Lu-DOTA^0^-Tyr^3^-octreotate, Somatostatin receptor scintigraphy, Phase I

## Abstract

**Purpose:**

Peptide receptor radionuclide therapy (PRRT) with ^177^Lu-DOTA^0^-Tyr^3^-octreotate (^177^Lu-DOTATATE) is one of the most reliable treatments for unresectable, progressive neuroendocrine tumors (NETs) with somatostatin receptor expression. We have, for the first time, reported the results of the tolerability, safety, pharmacokinetics, dosimetry, and efficacy of this treatment for Japanese patients with NET.

**Methods:**

Patients with unresectable, somatostatin receptor scintigraphy (SRS)-positive NETs were enrolled in this phase I clinical trial. They were treated with 29.6 GBq of ^177^Lu-DOTATATE (four doses of 7.4 GBq) combined with amino acid solution infusion plus octreotide long-acting release (LAR) 30 mg. The primary objective of this study was to evaluate the tolerability, safety, pharmacokinetics, and dosimetry of a single administration of this treatment in patients with SRS-positive NETs.

**Results:**

Six Japanese patients (three men and three women; mean age 61.5 years; range 50–70 years) with SRS-positive unresectable NETs were recruited. ^177^Lu-DOTATATE was eliminated from the blood in a two-phase manner. Cumulative urinary excretion of radioactivity was 60.1% (range 49.0%–69.8%) within the initial 6 h. The cumulative renal absorbed dose for 29.6 GBq of ^177^Lu-DOTATATE was 16.8 Gy (range 12.0–21.2 Gy), and the biological effective dose was 17.0 Gy (range 12.2–21.5 Gy). Administration of ^177^Lu-DOTATATE was well tolerated, with no dose-limiting toxicities. Grade 3 lymphopenia occurred in two (33.3%) cases, but there were no other severe toxicities. Four patients achieved partial response (objective response rate, 66.7%), one patient had stable disease, and one patient had progressive disease.

**Conclusion:**

PRRT with ^177^Lu-DOTATATE was well-tolerated and showed good outcomes in Japanese patients with unresectable NETs. Peptide receptor radionuclide therapy, ^177^Lu-DOTA^0^-Tyr^3^-octreotate .

**Supplementary Information:**

The online version contains supplementary material available at 10.1007/s12149-021-01674-9.

## Introduction

Neuroendocrine tumors (NETs) are rare, relatively indolent, and heterogeneous malignant diseases with predominantly neuroendocrine differentiation that can develop in any place of the human body [1]. Surgical resection is the most reliable treatment for patients with localized disease. However, these tumors are often diagnosed at an advanced stage of metastatic disease because of the nonspecific symptoms [2]. Currently approved systemic treatment for unresectable NETs include the mammalian target of rapamycin (mTOR) inhibitor everolimus, the tyrosine kinase inhibitor sunitinib, and the cytotoxic agent streptozotocin. Everolimus prolongs progression-free survival (PFS) in patients with unresectable NETs, sunitinib is approved for the treatment of unresectable pancreatic NET, and streptozotocin is authorized for pancreatic and gastrointestinal NETs in Japan [3–6].

Somatostatin analogs, such as octreotide long-acting release (LAR) and lanreotide, are effective treatment options for controlling tumor progression and hormonal function in patients with NETs [7, 8]. Radiolabeled somatostatin analogs are important imaging and therapeutic options. Somatostatin-based peptide receptor radionuclide therapy (PRRT) was introduced in the 1990s in Europe [9, 10]. Since then, it has developed into a valuable therapeutic tool for patients with NETs.

Recently, a prospective randomized phase III study (NETTER-1 study) has demonstrated superior outcomes in terms of longer progression-free survival (PFS; not reached vs. 8.4 months; hazard ratio 0.21) and overall survival (not reached vs. not reached; hazard ratio 0.40) after PRRT with ^177^Lu-DOTA^0^-Tyr^3^-octreotate (^177^Lu-DOTATATE) plus 30 mg octreotide LAR every 4 weeks compared with those after high-dose octreotide LAR (60 mg every 4 weeks) in patients with advanced midgut NETs, all progressed on regular doses of octreotide LAR [11, 12]. Therefore, the US Food and Drug Administration has approved ^177^Lu-DOTATATE for the treatment of somatostatin receptor (SSTR)-positive gastroenteropancreatic (GEP) NETs, including foregut, midgut, and hindgut NETs. The European Medicines Agency and the European Commission have also approved ^177^Lu-DOTATATE for the treatment of unresectable or metastatic, progressive, well-differentiated, SSTR-positive GEP-NETs. However, in Japan, PRRT with ^177^Lu-DOTATATEis not well established yet. Here we report the results from the first phase I study of PRRT for Japanese patients with unresectable NETs in Japan. The primary objective of this study was to evaluate the safety, tolerability, pharmacokinetics, and dosimetry of a single intravenous infusion of ^177^Lu-DOTATATE in combination with amino acid solution infusion and octreotide LAR.

## Materials and methods

### Study design

This study was conducted as an open-label, uncontrolled, single-center, phase I clinical study. PRRT with ^177^Lu-DOTATATE in combination with amino acid solution infusion and octreotide LAR was performed in patients with SSTR-positive, unresectable, progressive pancreatic, gastrointestinal, or pulmonary NETs to evaluate its tolerability, safety, pharmacokinetics, dosimetry, and efficacy. The presence of SSTRs on all target lesions documented by CT/MRI scans (RECIST Version 1.1 criteria) was evaluated based on positive somatostatin receptor scintigraphy (SRS) within 4 months prior to the study enrollment. The tumor uptake observed in each target lesion must be equal to or higher than the normal liver uptake observed on planar imaging according to the modified Krenning scale [13].

The primary objective of this study was to evaluate the safety, tolerability, pharmacokinetics, and dosimetry of a single intravenous infusion of ^177^Lu-DOTATATE at 7.4 GBq in combination with amino acid solution infusion and octreotide LAR. The secondary objectives of this study were to evaluate the safety of ^177^Lu-DOTATATE administration up to 29.6 GBq (four doses of 7.4 GBq) in combination with amino acid solution infusion and to evaluate the objective response rate (ORR).

This study was sponsored and designed by Fujifilm Toyama Chemical Co., Ltd. The study was conducted in accordance with the rules and principles of the 1964 Declaration of Helsinki, its subsequent amendments, Good Clinical Practice guidelines, and the manual on the proper use of lutetium-177-labeled somatostatin analog [16]. The study was approved by the Institutional Review Board of Yokohama City University Hospital and all participants provided signed informed consent.

## Patients

We planned to enroll six patients with unresectable, well-differentiated NETs who received currently approved systemic treatment and underwent SRS before PRRT.

The main inclusion criteria were histologically proven NETs with gastrointestinal, pancreatic, or lung origin; Ki67 index ≤ 20%; the presence of at least one measurable lesion according to the RECIST criteria; the presence of SSTR on all target lesions evaluated based on positive SRS; serum creatinine level ≤ 1.7 mg/dL, creatinine clearance ≥ 50 mL/min, hemoglobin level ≥ 8.0 g/dL, WBC count ≥ 2 × 10^9^/L (2 × 10^3^/mm^3^), platelet count ≥ 75 × 10^9^/L (7.5 × 10^4^/mm^3^), total bilirubin level ≤ 3 × ULN, serum albumin level > 3.0 g/dL.

The main exclusion criteria were pathologically poorly-differentiated NETs, neuroendocrine carcinoma, small cell carcinoma, and large cell carcinoma; any surgery, radioembolization, chemoembolization, and radiofrequency ablation within 12 weeks prior to enrollment in the study; administration of everolimus, sunitinib, streptozotocin, or other systemic cytotoxic agents within 8 weeks prior to enrollment; pregnancy or lactation; female patients of child-bearing potential or male patients with a female partner of child-bearing potential who cannot agree to use contraceptive methods until 6 months after the last administration of ^177^Lu-DOTATATE; receiving treatment with short-acting octreotide, which cannot be interrupted for 24 h before and 4 h after each administration of ^177^Lu-DOTATATE or receiving treatment with octreotide LAR, which cannot be interrupted for at least 6 weeks before the administration of ^177^Lu-DOTATATE according to NETTER-1 study protocol; prior external beam radiation therapy administered to ≥ 25% of the bone marrow.

### Treatment schedule

Four administrations of 7.4 GBq (± 10%) ^177^Lu-DOTATATE were administered concomitantly with amino acid solution. ^177^Lu-DOTATATE was administered over 30 min. The amino acid solution was administered by intravenous infusion at approximately 1000 mL over 4 h starting 30 min before ^177^Lu-DOTATATE administration. It was confirmed that the concomitant use of an amino acid solution infusion containing 2.5% lysine and 2.5% arginine promoted urinary excretion of ^177^Lu-DOTATATE, reducing the absorbed dose to the kidney by 47% on average [17]. A dose of 30 mg of octreotide LAR was injected the next day. ^177^Lu-DOTATATE was administered at 8 ± 1-week intervals, which could be extended up to 16 weeks to resolve acute toxicity. In cases where patients experienced clinical symptoms associated with their functional NETs, octreotide s.c rescue injections were allowed.

Before initiating the amino acid solution infusion, an intravenous bolus of appropriate antiemetics was administered to relieve and prevent nausea and vomiting due to the infusion. Antiemetics typically used to prevent nausea due to anticancer therapy (serotonin [5-HT3] receptor antagonists such as granisetron and ondansetron) were used. When nausea/vomiting was not alleviated using the injectable alone, additional antiemetics including oral agents were administered.

### Evaluations

Safety was evaluated based on adverse events, laboratory tests, vital signs, physical examination, arterial oxygen saturation (SpO_2_), 12-lead electrocardiogram (ECG), body weight, and Eastern Cooperative Oncology Group (ECOG) performance status. Toxicity was evaluated according to the NCI CTCAE criteria version 4.0 14). Blood count and liver and kidney biochemistry examinations were performed just prior to the treatment and 5 days, 2, 4, 6, and 8 weeks after the first administration (Course 1). In addition, the examinations were performed the second, third, fourth administration (Courses 2, 3, 4), and as per routine clinical follow-up thereafter.

Course 2 treatment and subsequent courses were initiated for subjects who completed the dose-limiting toxicity (DLT) observation period (up to Day 56 of Course 1) without experiencing DLT and who met the eligibility criteria. DLT was defined as grade ≥ 4 neutropenia with fever (> 38.3 °C), anemia with blood transfusion, grade ≥ 3 platelet count decreased requiring transfusion, grade 4 thrombocytopenia that lasts for seven days, grade 3 or 4 serum creatinine elevation with creatinine clearance decreased over 40% compared with base line, grade 3 or 4 non-hematological toxicities with serum liver enzyme (aspartate aminotransferase, ALT; alanine aminotransferase, AST; alkaline phosphatase, ALP; and gamma-glutamyl transpeptidase, γGTP) elevation over two levels, and grade 3 or 4 non-hematological toxicities that lasts for two weeks. The study treatment was considered tolerable when the number of subjects who experienced DLT was zero or one per six subjects. The appropriateness of DLT, as assessed by the investigator or sub-investigator, was confirmed by the clinical trial medical expert. All adverse events and serious adverse events, reported spontaneously or non-spontaneously, were collected during the study.

Objective tumor response was assessed every 12 weeks from the first treatment date and continued until 24 weeks after the last administration of ^177^Lu-DOTATATE (Day 169 of the last course) according to RECIST Version 1.1 criteria [15]. The ORR was defined as the proportion of patients with complete response (CR) plus partial response (PR). In addition to assessments performed by the investigator or sub-investigator, an independent central review by the reading committee was also conducted.

Blood and urine collection for pharmacokinetic evaluation, and ^177^Lu-DOTATATE imaging for dosimetry evaluation were performed during Days 1–7 of Course 1. Image processing and radioactivity quantification were performed by a nuclear medicine expert at site, and dosimetry calculation was performed by the sponsor of the study. All images were taken using the Symbia T-16 SPECT/CT scanner (Siemens). The dual-headed gamma camera was equipped with a 0.952-cm (0.375-in)-thick NaI crystal and a medium-energy low penetration collimator. An energy window of ± 10% was applied around the one dominant gamma ray energies of ^177^Lu (208 keV). The matrix size was 256 × 1024. Anterior and posterior whole-body scanning at a scan speed of 10 cm/min was performed 1, 4, 24, 48 h and 120 h after administration of ^177^Lu-DOTATATE. Radioactivity quantification was performed on patient images, and the geometric mean was calculated. Regions of interests (ROIs) were drawn around the kidneys, liver, spleen, other organs, and whole body on the planar images. Tumor tissue within the organs was excluded. The absorbed dose to each organ was estimated using the OLINDA/EXM 1.0 (Vanderbilt University), a software based on the Medical Internal Radiation Dose algorithm, which is widely used for established beta and gamma emitting radionuclides. The biological effective dose (BED) for the kidneys were estimated using the SAS software, version 9.4 (SAS Institute).

### Statistical analysis

Statistical analysis was performed using the SAS software, version 9.4 (SAS Institute). Continuous variables are presented as means, standard deviations, medians and ranges as appropriate, and categorical variables are presented as counts and percentages. The 90% confidence interval for ORR was calculated with the Clopper-Pearson’s exact method. Since the sample size of this study was small, it was expected that if the level of confidence interval was set as 95%, the width of confidence interval would be large and it would be difficult to evaluate the profile of efficacy. In addition, the efficacy evaluation in this study was exploratory. Therefore, we adopted the 90% confidence interval to evaluate the efficacy.

## Results

### Patients

Between August 2017 and May 2019, six Japanese patients were enrolled in the study (mean age 61.5 years; range 50–70 years). There were three men and three women. ECOG Performance Status was 0 in all patients. All patients had multiple liver metastatic neuroendocrine tumors, and five (83.3%) patients underwent surgical resection for primary lesions. The primary tumor lesions were located in the pancreas (*n* = 3), rectum (*n* = 2), and stomach (*n* = 1). Two patients had a history of splenectomy with distal pancreatectomy due to primary pancreatic NET. According to the WHO 2017/2019 classification, all patients were classified as having grade 2 disease (Ki67 Index; mean 8.4%; range 3%–14.0%). The median duration from diagnosis to initial PRRT was 59.6 months (range 5.9–314 months). The median number of previous systemic treatment was 1 (range 1–3). Krenning’s scale score of the most recent SRS was grade 4 (very intense uptake, >  > kidneys and spleen) in five cases and grade 3 (intense uptake, > liver) in one case. The patient characteristics are shown in Table 1.

**Table 1:** Patient demographics, treatment, and tumor characteristics in patients with NETs (*n* = 6)

Sex, *n* (%)	
Male	3 (50)
Female	3 (50)
Age (years), mean (range)	61.5 (50–70)
Primary tumor site, *n* (%)	
Pancreas	3 (50)
Stomach	1 (16.7)
Rectum	2 (33.3)
Site of metastasis, *n* (%)	
Liver	6 (100)
Lymph nodes	1 (16.7)
Bone	1 (16.7)
Lungs	0 (0)
Pathological classification	
NET Grade 1	0
NET Grade 2	6 (100)
Ki67 labeling index, (%) mean (range)	8.4 (3–14.0)
SRS, Krenning scale, *n* (%)	
Grade 2	0 (0)
Grade 3	1 (16.7)
Grade 4	5 (83.3)
Period from diagnosis to PRRT (months) median (range)	59.6 (5.9–314)
Previous systemic treatment number median (range)	1 (1–3)
Previous surgical resection	5 (83.3)
Previous treatment	6 (100)
Somatostatin analog	
Previous treatment	1 (16.7)
Molecular targeted therapy	
Previous treatment	1 (16.7)
Chemotherapy	

*NET* neuroendocrine tumor, *SRS* somatostatin receptor scintigraphy

### Drug presence and elimination from the blood

**Fig. 1:**
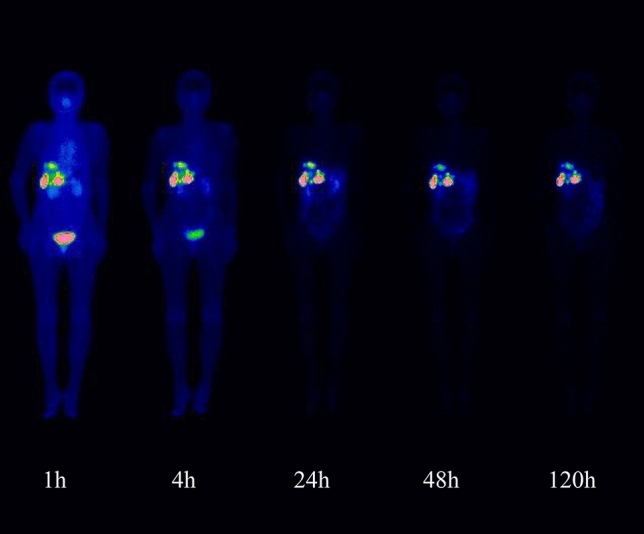
This figure illustrates the biodistribution/gamma camera images of [177Lu-DOTA^0^, Tyr^3^] octreotate over time. Multiple liver metastasis lesions showed intense uptake at 1 h and the uptake gradually decreased with time

**Fig. 2:**
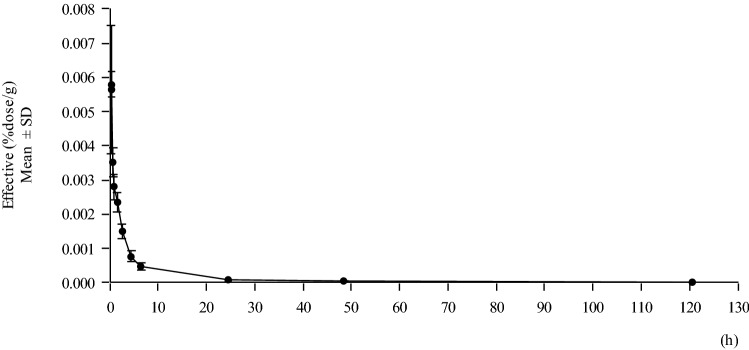
Mean blood radioactivity concentration over time. 177Lu was eliminated from the blood in a two-phasic manner according to non-compartment model analysis. Effective half time of [177Lu-DOTA^0^, Tyr^3^]octreotate was 2.37 ± 0.30 h (mean ± SD) in distribution phase and 42.7 ± 2.44 h (mean ± SD) in excretion phase

Figure 1 shows the biodistribution/gamma camera images of ^177^Lu-DOTATATE over time. The median duration of treatment was 56 days (range 51–76 days), and the number of injections was four. The median cumulative dose of ^177^Lu-DOTATATE was 29.4 GBq. All patients received concomitant amino acid solution infusion with each administration. After ^177^Lu-DOTATATE injection, radioactivity was eliminated from the blood in a two-phase manner according to the non-compartment model analysis (Fig. 2). Effective half-time of ^177^Lu-DOTATATE (mean ± SD) was 2.37 ± 0.30 h in the distribution phase and 42.7 ± 2.44 h in the excretion phase. The fraction of the injected activity retained in the blood at 20 min after the injection was mean 0.00351% dose/g (range 0.00272%–0.00382% dose/g), while that at 4 h after the injection was mean 0.000770% dose/g (range 0.000537–0.000985% dose/g).

### Excretion

The effective urinary radioactivity concentration (mean ± SD) was 4.7 ± 1.9 GBq/L in the first hour and gradually decreased to 0.3 ± 0.1 GBq/ L in the first 24 h (Table 2).

**Table 2:** Urinary radioactivity concentration (*n* = 6)

	Effective urinary radioactivity concentration (GBq/L) mean ± SD
0–1 h	4.7 ± 1.9
1–4 h	1.7 ± 0.7
4–6 h	1.3 ± 1.1
6–24 h	0.3 ± 0.1
24–48 h	0.07 ± 0.03

**Fig. 3:**
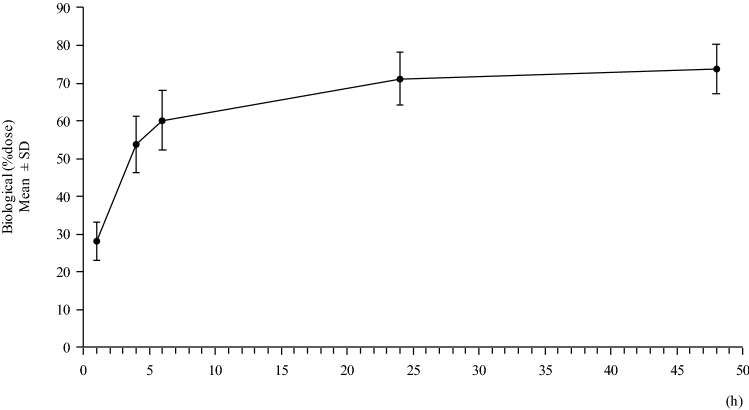
Cumulative urinary excretion of radioactivity. The mean biological cumulative urine excretion of radioactivity reached 53.7% (range 42.6–61.7%) in the initial 4 h and reached 71.2% (range 62.5–81.4%) at 24 h after administration

The mean biological cumulative urine excretion of radioactivity reached 53.7% (range 42.6%–61.7%) in the first 4 h and reached 71.2% (range 62.5%–81.4%) at 24 h after administration (Fig. 3).

### Biodistribution

**Fig. 4:**
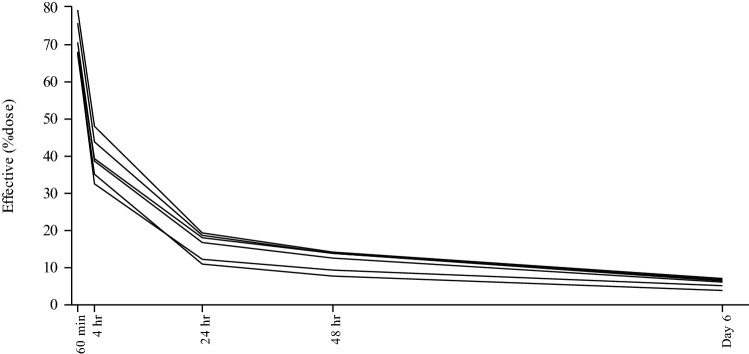
The fraction of the effective radioactivity of ^177^Lu-DOTATATE retained in the whole body at 1 h after a single injection was 71.4 ± 4.9% (mean ± SD), which decreased to 39.5 ± 5.7% (mean ± SD) at 4 h, 16.0 ± 3.5% (mean ± SD) at 24 h, and 5.8 ± 1.3% (mean ± SD) at Day 6 (Fig. 4)

The fraction of the effective radioactivity of ^177^Lu-DOTATATE (mean ± SD) retained in the whole body at 1 h after a single injection was 71.4 ± 4.9%, which decreased to 39.5 ± 5.7% at 4 h, 16.0 ± 3.5% at 24 h, and 5.8 ± 1.3% at Day 6 (Fig. 4). Effective radioactivity in each organ at 1 h after a single injection was 4.97 ± 2.01% in the liver, 2.39 ± 0.535% in the kidney, and 0.812 ± 0.405% in the spleen, and then gradually decreased (Supplements 1, 2, and 3).

### Radiation dosimetry

The mean absorbed doses to each organ in the non-splenectomized four patients are presented in Table 3. The majority of the radiation dose from ^177^Lu-DOTATATE was found in the kidney, spleen, urinary bladder wall, and liver. The calculated absorbed doses (mean ± SD) to the kidney, spleen, urinary bladder wall, red bone marrow, and liver were 0.568 ± 0.128, 0.559 ± 0.273, 0.463 ± 0.126, 0.024 ± 0.003, and 0.247 ± 0.180 mGy/MBq, respectively. The BED of the kidney was 0.574 ± 0.131 mGy/MBq and 17.0 ± 3.9 Gy/ 29.6 GBq (Table 4).

**Table 3:** Absorbed dose for target organ (non-splenectomized four patients)

Target organ	*n*	Mean	SD	Mean	SD
Absorbed dose		mGy/MBq		Gy/29.6 GBq	
Brain	4	0.019	0.002	0.566	0.067
Lungs	4	0.020	0.002	0.613	0.051
Breasts	4	0.019	0.002	0.568	0.057
Liver	4	0.247	0.180	7.31	5.33
Spleen	4	0.559	0.273	16.5	8.08
Stomach wall	4	0.022	0.002	0.644	0.067
Small intestine	4	0.021	0.002	0.633	0.059
Upper large intestine wall	4	0.022	0.002	0.639	0.054
Lower large intestine wall	4	0.021	0.002	0.630	0.065
Kidneys	4	0.568	0.128	16.8	3.80
Urinary bladder wall	4	0.463	0.126	13.7	3.74
Red bone marrow	4	0.024	0.003	0.722	0.088
Whole body	4	0.031	0.003	0.914	0.090

Target organ	*n*	Mean	SD	Mean	SD
		mSv/MBq		Sv/29.6 GBq	
Effective dose equivalent	4	0.128	0.015	3.79	0.440
Effective dose	4	0.066	0.005	1.95	0.155

*SD* standard deviation

**Table 4:** Biological effective dose (BED) for the kidneys (subjects without spleen excision)

	*n*	Mean	SD	Median	Min	Max
BED (mGy/MBq)	4	0.574	0.131	0.580	0.411	0.727
BED for 7.4 GBq (Gy/7.4 GBq)	4	4.25	0.973	4.29	3.04	5.38
BED for 29.6 GBq (Gy/29.6 GBq)	4	17.0	3.89	17.2	12.2	21.5

*BED* biological effective dose, *SD* standard deviation

### Safety

Treatment-emergent adverse events (TEAEs) are summarized in Table 5. The most common non-hematological adverse events were diarrhea (83.3%), malaise (66.7%), decreased appetite (66.7%), nausea (50%), abdominal distension (50%), and alopecia (50%). There were no severe (grade ≥ 3) adverse events associated with non-hematological toxicities. The common hematological adverse events were lymphopenia (50%), leukopenia (33.3%), and thrombocytopenia (33.3%). Severe hematological TEAEs that occurred were only grade 3 lymphopenia (33.3%).

**Table 5:** Treatment-emergent adverse events by severity

System organ class (SOC)^*1^		Grade 1	Grade 2	Grade 3	Grade 4
	Preferred term (PT)	*n* (%)	*n* (%)	*n* (%)	*n* (%)
All treatment emergent adverse events (TEAEs)		0	4 (66.7)	2 (33.3)	0
Blood and lymphatic system disorders		0	1 (16.7)	2 (33.3)	0
	Leukopenia	0	2 (33.3)	0	0
	Lymphopenia	0	1 (16.7)	2 (33.3)	0
	Thrombocytopenia	0	2 (33.3)	0	0
Gastrointestinal disorders		4 (66.7)	2 (33.3)	0	0
	Abdominal distension	3 (50.0)	0	0	0
	Abdominal pain	1 (16.7)	0	0	0
	Constipation	2 (33.3)	0	0	0
	Diarrhea	3 (50.0)	2 (33.3)	0	0
	Nausea	3 (50.0)	0	0	0
General disorders and administration site conditions		5 (83.3)	0	0	0
	Chest discomfort	1 (16.7)	0	0	0
	Chest pain	1 (16.7)	0	0	0
	Malaise	4 (66.7)	0	0	0
	Pyrexia	1 (16.7)	0	0	0
Immune system disorders		1 (16.7)	0	0	0
	Seasonal allergy	1 (16.7)	0	0	0
Infections and infestations		2 (33.3)	1 (16.7)	0	0
	Gingivitis	1 (16.7)	0	0	0
	Nasopharyngitis	2 (33.3)	0	0	0
	Oral herpes	0	1 (16.7)	0	0
Investigations		1 (16.7)	4 (66.7)	0	0
	ALT increased	1 (16.7)	0	0	0
	AST increased	1 (16.7)	0	0	0
	Cholesterol increased	0	1 (16.7)	0	0
	CPK increased	0	1 (16.7)	0	0
	Creatinine increased	1 (16.7)	0	0	0
	γGTP increased	0	2 (33.3)	0	0
Metabolism and nutrition disorders		3 (50.0)	2 (33.3)	0	0
	Hyperuricemia	1 (16.7)	0	0	0
	Hypocalcemia	0	1 (16.7)	0	0
	Decreased appetite	3 (50.0)	1 (16.7)	0	0
Musculoskeletal and connective tissue disorders		1 (16.7)	0	0	0
	Musculoskeletal pain	1 (16.7)	0	0	0
Nervous system disorders		3 (50.0)	1 (16.7)	0	0
	Dizziness	1 (16.7)	0	0	0
	Dysgeusia	0	1 (16.7)	0	0
	Headache	1 (16.7)	0	0	0
	Taste disorder	1 (16.7)	0	0	0
Skin and subcutaneous tissue disorders		3 (50.0)	1 (16.7)	0	0
	Alopecia	3 (50.0)	0	0	0
	Dyshidrotic eczema	0	1 (16.7)	0	0
	Rash	1 (16.7)	0	0	0

*1 Adverse event term: MedDRA, version 22.0

*TEAE* treatment-emergent adverse events, *ALT* alanine aminotransferase, *AST* aspartate aminotransferase, *CPK* creatine phosphokinase, *γGTP* gamma-glutamyl transferase

Lymphocyte, erythrocyte, and leukocyte counts gradually decreased after each treatment; however, there were no remarkable changes during the follow-up period (Supplements 4, 5, and 6). Serum creatinine clearance levels also showed no remarkable change during the follow-up period (Supplement 7).

There were no severe infections, no other severe toxicities, no secondary hematological malignant diseases, and no DLT. No adverse events related to the amino acid solution infusion were observed.

### Efficacy

Efficacy was evaluated in all six patients who received the treatment (Table 6). For both the site reading and the central reading, the ORR was 66.7% (90% CI 27.1–93.7). CR was observed in none of the patients, PR was observed in four patients, SD was observed in one patient, and progressive disease was observed in one patient. ORR in pancreatic NET and gastrointestinal NET was 66.7% and 66.7%, respectively. In all cases, including the progressive disease case, the total diameter of the target lesions decreased in size (Supplement 8).

**Table 6:** Objective response rate (central assessment)

Parameter	Number of subjects (*n* = 6) (%)
CR	0
PR	4 (66.7)
SD	1 (16.7)
Non-CR/non-PD	0
PD	1 (16.7)
NE	0
ORR (CR + PR)	4 (66.7)
Two-sided 90% confidence interval^*1^	27.1–93.7

*CR* complete response; *PR* partial response; *SD* stable disease; *PD* progressive disease; *NE* no evaluated; *ORR* objective response rate

^*1^Clopper-Pearson’s exact confidence interval (%)

## Discussion

This study is the first clinical trial on ^177^Lu-DOTATATE for unresectable NETs in Japan. At 29.6 GBq, the absorbed dose to the kidney and red bone marrow was 16.8 Gy and 0.722 Gy, respectively. These values were lower than the reported threshold dose for the kidney (23 Gy) and the red bone marrow (2 Gy) [18, 19]. The calculated absorbed doses to the kidney, spleen, red bone marrow, and liver were 0.568, 0.559, 0.0244 and 0.247 mGy/MBq, respectively, in this study. We compared the calculated absorbed dose to each organ with previously published studies in Caucasian populations. In the previous studies, the absorbed dose to the kidney was 0.88 and 0.68, to the spleen was 2.16 and 0.645, to the red bone marrow was 0.07 and 0.03, and to the liver was 0.22 and 0.245 mGy/Bq, respectively [20, 21]. The absorbed doses to the kidney, red bone marrow, and liver in previous studies were very similar to the data in our study. However, the absorbed dose to the spleen was not constant in previous studies, and the standard deviation was also relatively large in our study. The kidney is the dose-limiting organ for this agent. In this study, the blood creatinine level increased in only one case (16.7%) and its severity was grade 1. In the Erasmus study and the NETTER-1 study, severe renal toxicities were rare (1% and 0.9%, respectively) [11, 22]. Renal toxicity was suppressed by the concomitant use of an amino acid solution infusion. In a rat model, the quantification of ^177^Lu in the kidney was significantly reduced by approximately 45% in the kidneys of lysine-protected rats [17]. Competition for the megalin receptor by lysine and radiolabeled octreotate, containing l lysine residue, might explain the reduction in renal retention after coadministration with cationic amino acid [23].

In this study, severe lymphopenia occurred in two (33.3%) cases, and other hematological toxicities were grade 1 or 2 leukocytopenia and thrombocytopenia. This finding was very similar to that reported in the Erasmus study. Further, lymphopenia was the most common severe adverse event (50.0%) in the Erasmus study; however, at the follow-up visit after 3 month final treatment, the frequency of severe lymphopenia was 25.8%, and at the follow-up visit after 30 months final treatment, its frequency decreased to 5.6% [22]. In the present study, lymphocyte, erythrocyte, and leukocyte counts gradually decreased after each treatment; however, there was a slight increase of lymphocyte count, and severe lymphopenia decreased to 16.7% during the follow-up period. In the present study, mild gingivitis (*n* = 1), nasopharyngitis (*n* = 2), and oral herpes (*n* = 1) occurred, but there was no severe infection. In the NETTER-1 study, pancytopenia, lymphopenia, leukocytopenia, neutropenia, and thrombocytopenia were more common in the ^177^Lu-DOTATATE group than in the control group; however, there was no difference in terms of infection between the groups [11].

The most common non-hematological adverse events were diarrhea (83.3%), malaise (66.7%), decreased appetite (66.7%), nausea (50%), abdominal distension (50%), and alopecia (50%). The frequency of diarrhea in this study was slightly higher than that in the NETTER-1 study (83.3% vs. 28.8%) [11]. In this study, the primary lesions were located in the pancreas, stomach, and rectum. Therefore, many patients underwent pancreatectomy and colectomy before enrollment in the study; diarrhea is likely to occur more in such patients compared to primary midgut cases because pancreatic enzyme deficiency or rectal dysfunction often leads to diarrhea. It is also difficult to distinguish the cause of diarrhea ^177^Lu-DOTATATE from concomitant octreotide LAR, because diarrhea is not so rare side effect of somatostatin analog.

## Conclusion

PRRT with ^177^Lu-DOTATATE was well-tolerated and showed good outcomes in Japanese patients. This study may be a promising treatment option for Japanese patients with unresectable NETs.

## Supplementary Information

Below is the link to the electronic supplementary material.

Supplementary file1 (PPTX 78 KB)
