# Supplementary material for: Safety and efficacy of peptide receptor radionuclide therapy with 177Lu-DOTA0-Tyr3-octreotate in combination with amino acid solution infusion in Japanese patients with somatostatin receptor-positive, progressive neuroendocrine tumors
Source: Ann Nucl Med. 2021 Sep 17;35(12):1332–41. doi: 10.1007/s12149-021-01674-9 (PMC8557155; doi:10.1007/s12149-021-01674-9)
Supplement: Supplementary file 1 — Supplementary file1 (PPTX 78 KB) [file 12149_2021_1674_MOESM1_ESM.pptx]

## Slide 1
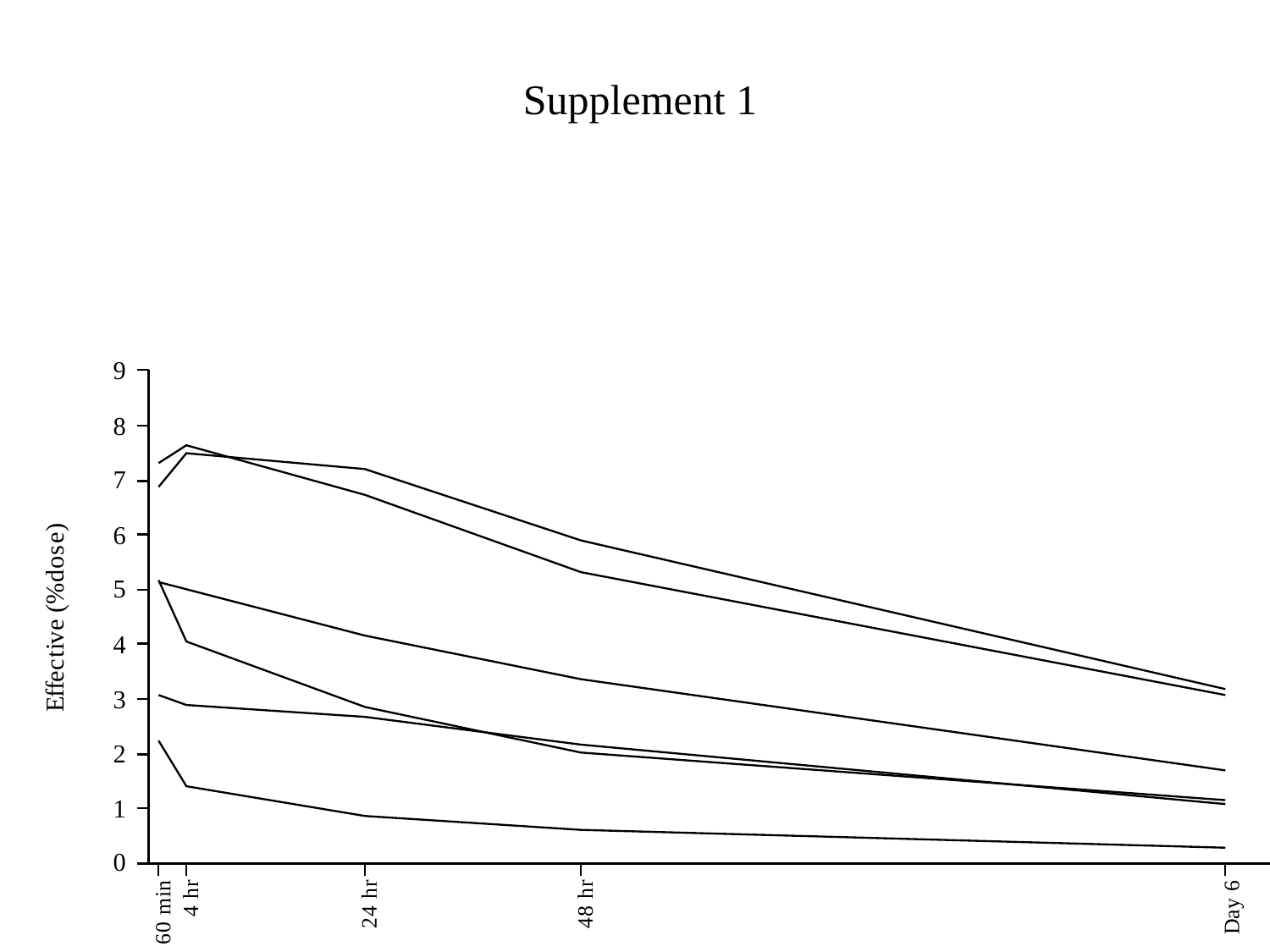

Supplement 1

## Slide 2
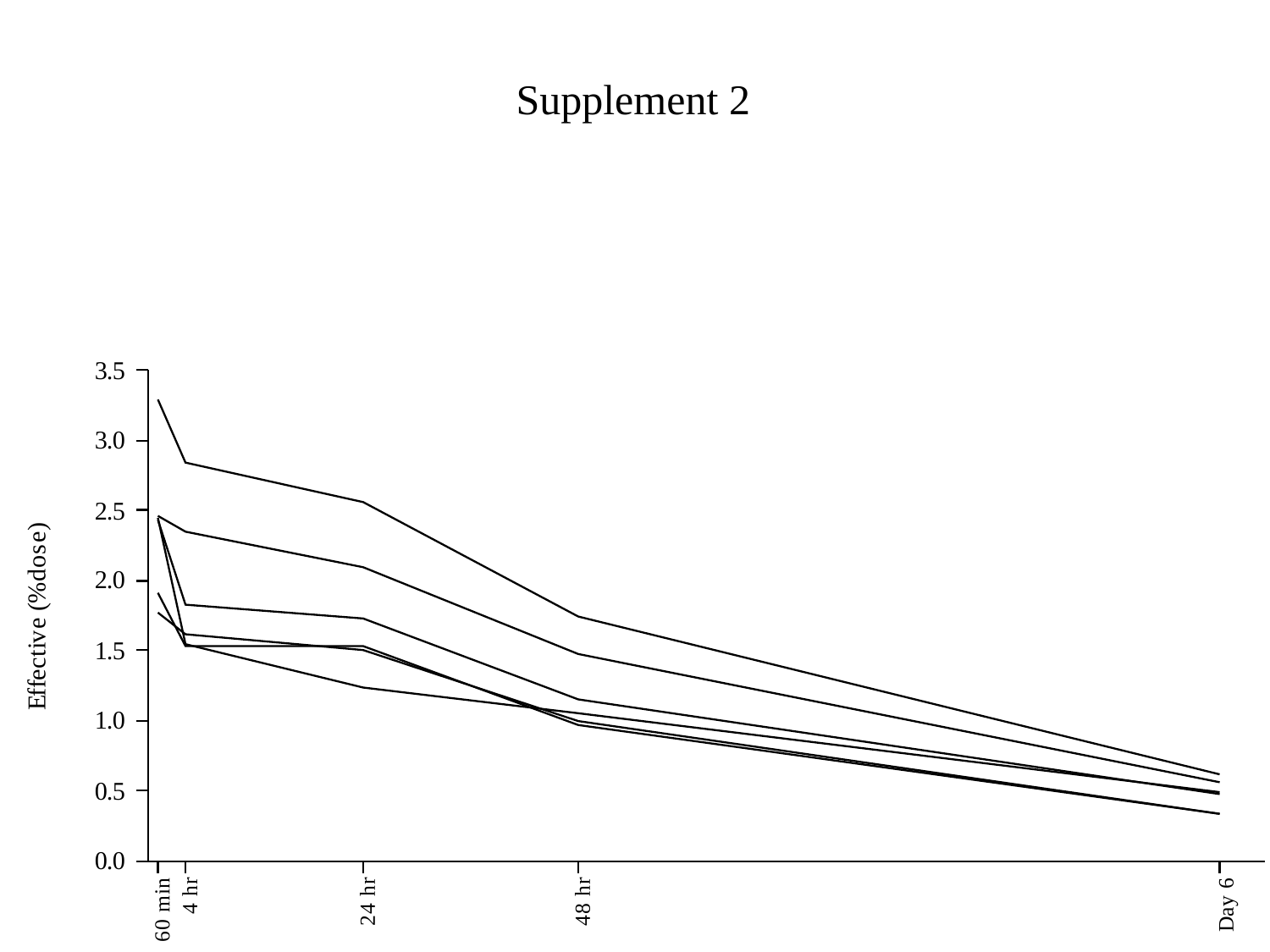

Supplement 2

## Slide 3
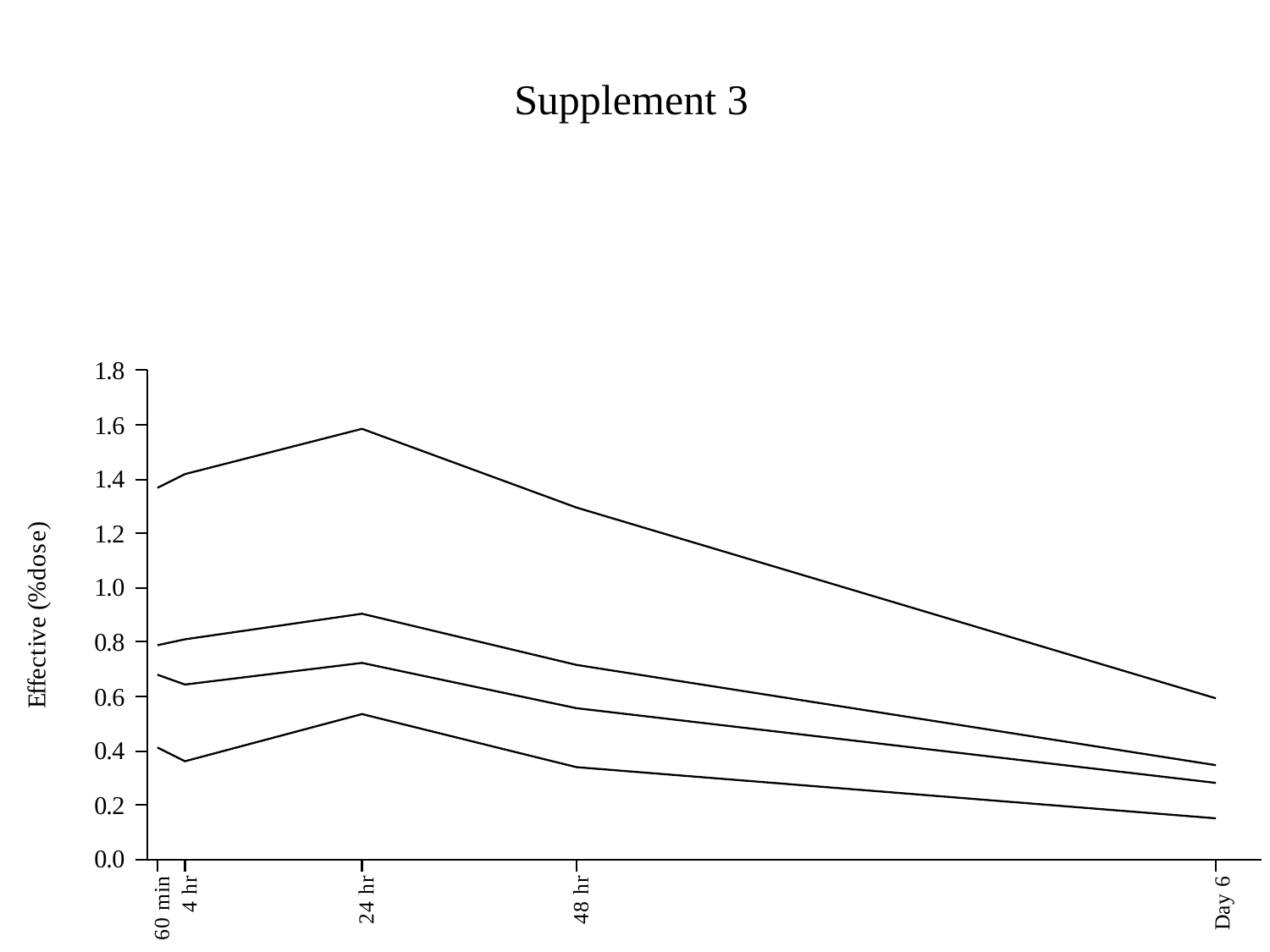

Supplement 3

## Slide 4
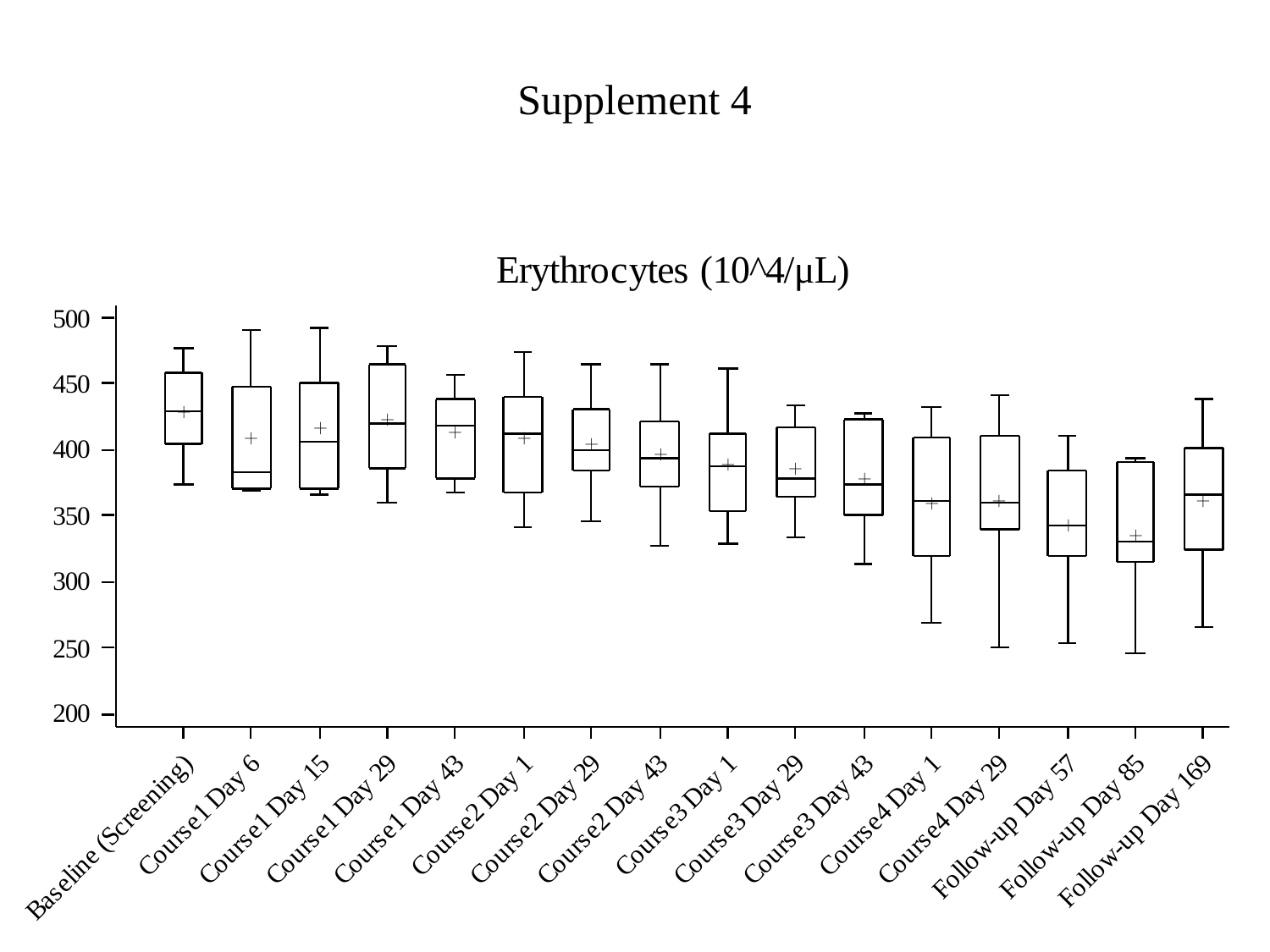

Supplement 4

## Slide 5
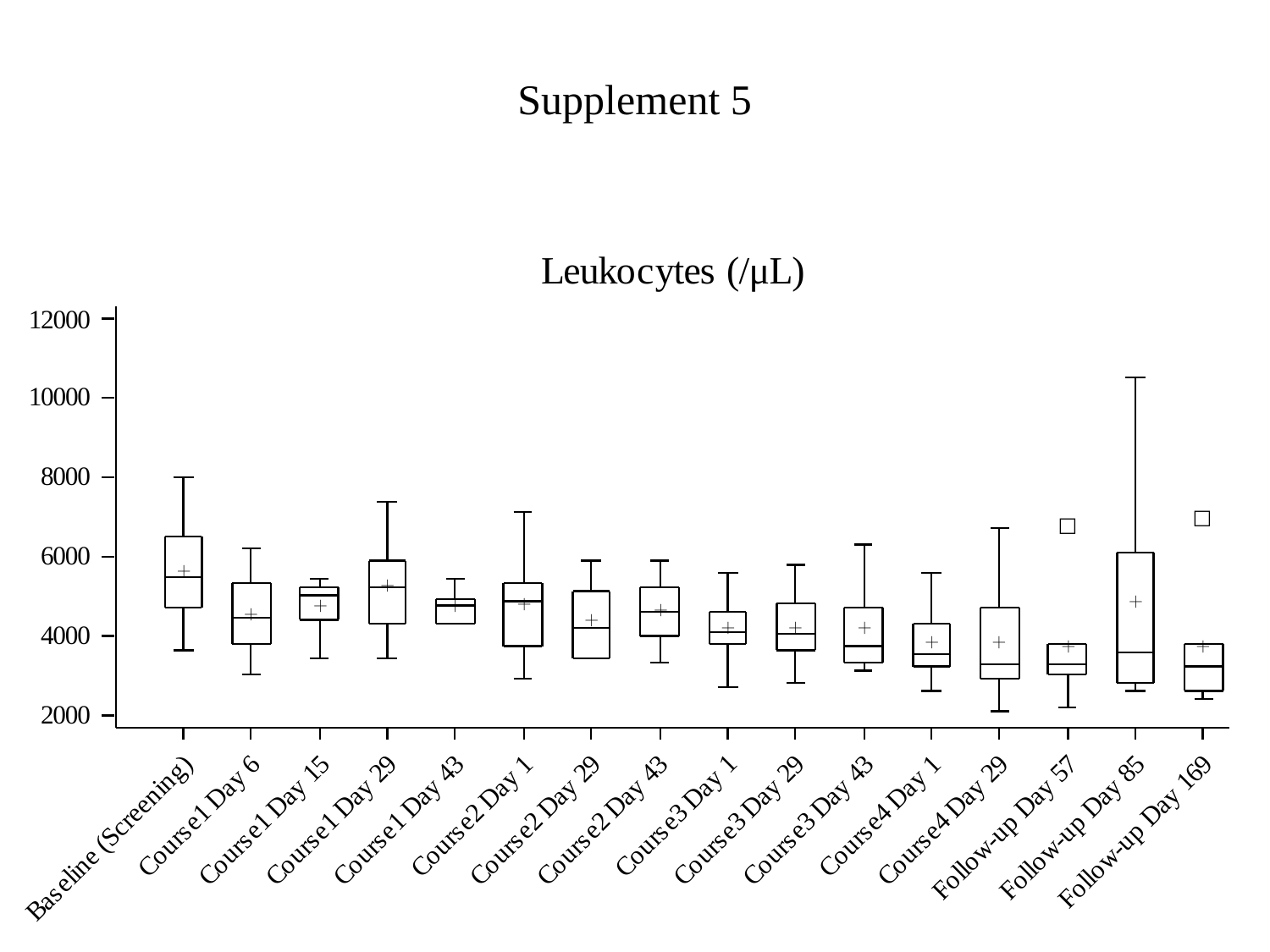

Supplement 5

## Slide 6
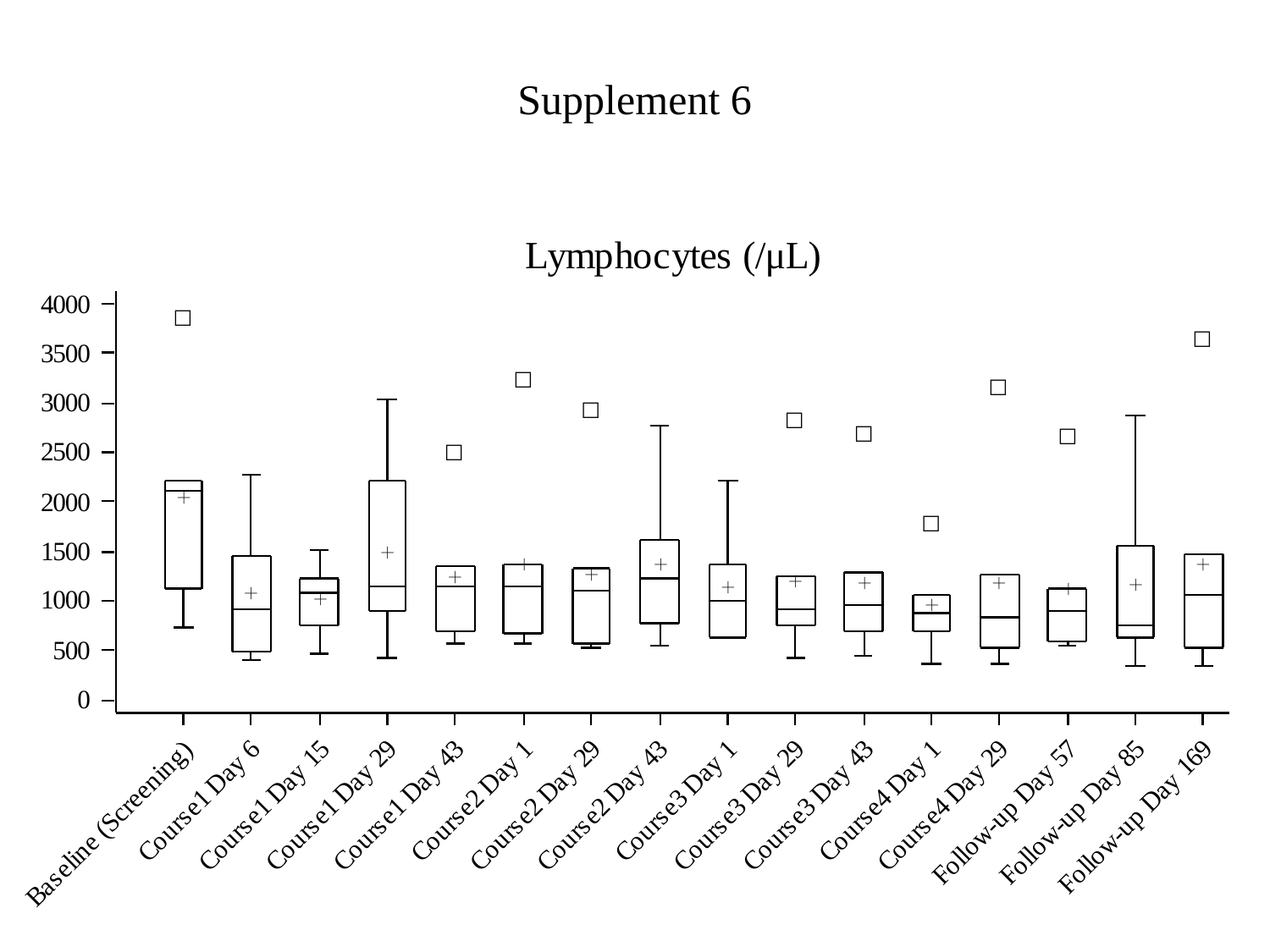

Supplement 6

## Slide 7
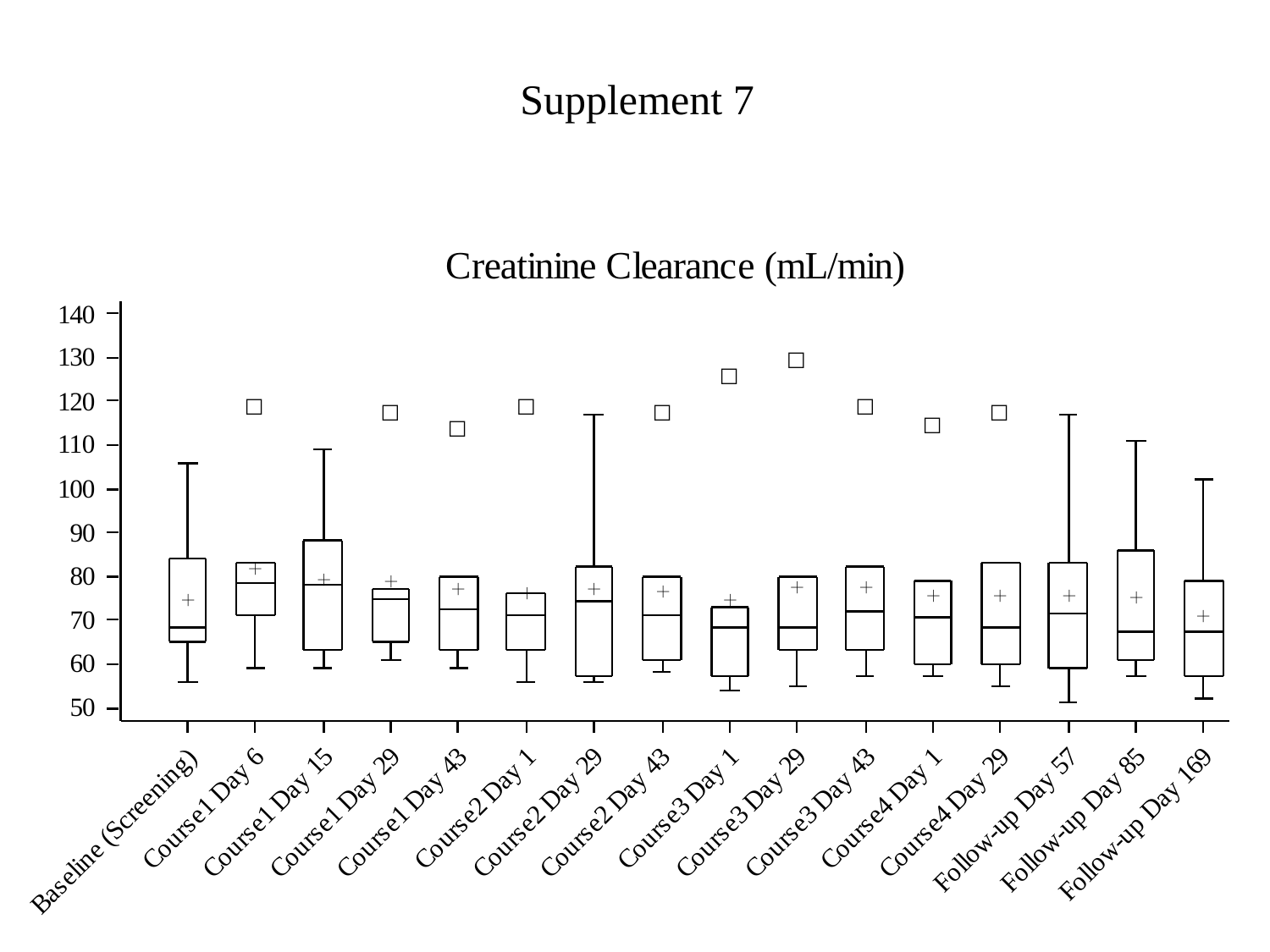

Supplement 7

## Slide 8
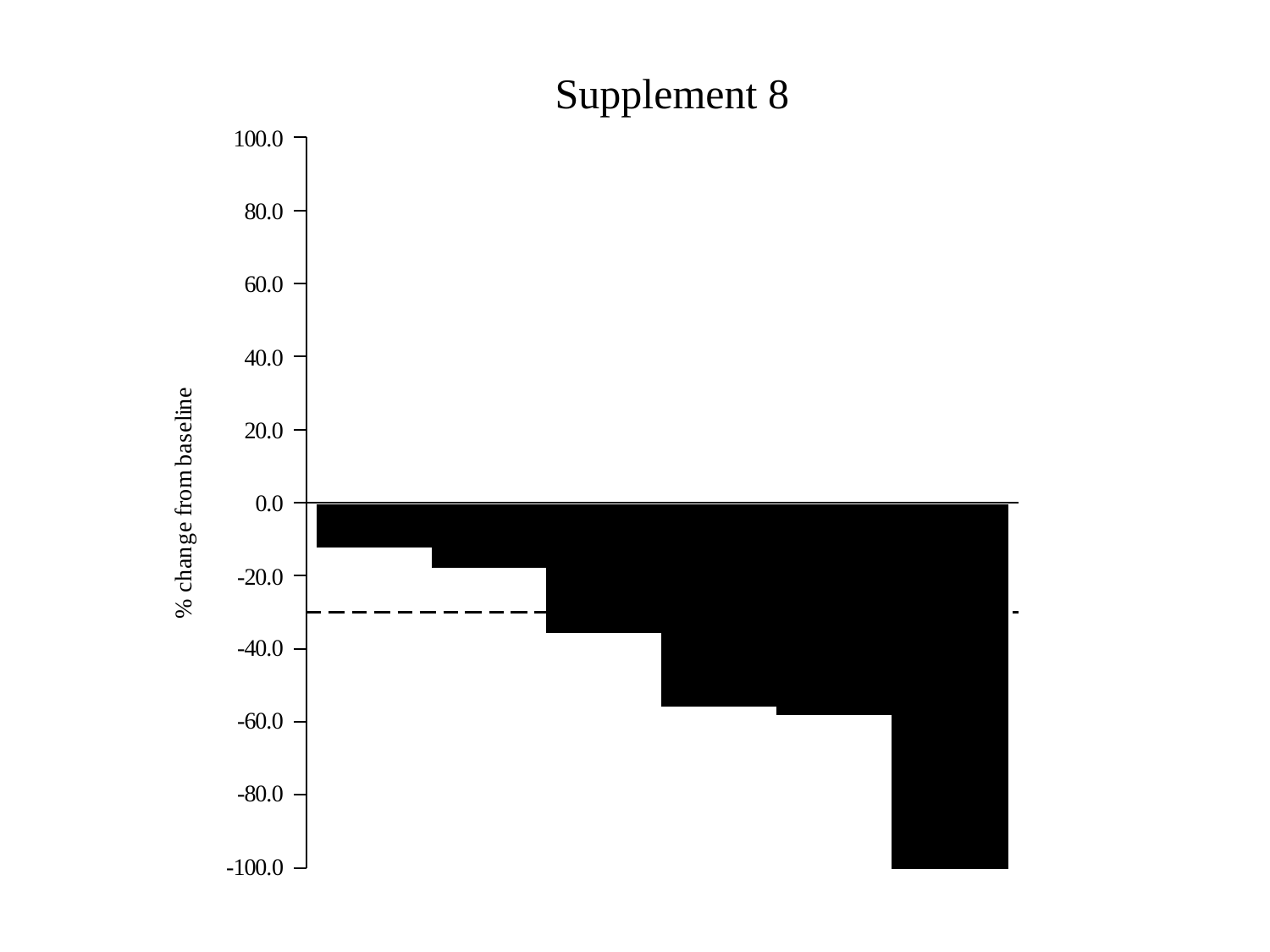

Supplement 8
